# How empowered are girls/young women in their sexual relationships? Relationship power, HIV risk, and partner violence in Kenya

**DOI:** 10.1371/journal.pone.0199733

**Published:** 2018-07-19

**Authors:** Julie Pulerwitz, Sanyukta Mathur, Daniel Woznica

**Affiliations:** 1 HIV and AIDS Program, Population Council, Washington, DC, United States of America; 2 Bloomberg School of Public Health, Johns Hopkins University, Baltimore, MD, United States of America; UNAIDS, UNITED STATES

## Abstract

**Background:**

Gendered power dynamics within couple relationships can constrain women from achieving positive sexual and reproductive health outcomes. But little is known about relationship power among adolescents, and tools to measure it are rarely validated among adolescents. We tested the Sexual Relationship Power Scale (SRPS) among adolescent girls and young women (AGYW) and examined associations with select health outcomes.

**Methods:**

A 16-item adaptation of the SRPS was administered to AGYW aged 15–24 in Kenya (n = 1,101). Confirmatory factor analysis (CFA) and theta coefficients assessed scale performance for three age bands: 15–17, 18–20, and 21–24 years old. Relationship power levels were examined and multivariate logistic regressions assessed the relationship between power, and partner violence and HIV risk outcomes.

**Results:**

CFAs confirmed a one factor structure for each subgroup, and thetas for final 15-item scales were robust (>.82). Most respondents reported limited power in their sexual relationships, however older respondents consistently reported lower levels of power. Relationship power was strongly associated with several outcomes, even when controlling for socioeconomic status and schooling. For example, AGYW who reported more relationship power were 12, 6, and 7 times less likely (ages 21–24, 18–20, and 15–17, respectively) to experience sexual violence (p<0.001). Significant relationships were also found in multivariate analyses for physical partner violence (all three age bands), using a condom at last sex (18–20-year-olds), and increased knowledge of partner’s HIV status (21–24-year-olds).

**Conclusions:**

The SRPS is a good measure of relationship power for several age bands within AGYW, and power is experienced differently by older and younger AGYW. Low relationship power was a consistent predictor of partner violence, as well as an important predictor of HIV risk. Interventions seeking to address HIV and violence should also explicitly address relationship power and utilize validated tools (like the SRPS) to evaluate impacts.

## Introduction

Numerous studies have shown that power imbalances within sexual relationships are linked to poor sexual and reproductive health (SRH) outcomes for women worldwide [1–7]. For instance, those with high levels of relationship power were five times as likely as women with low levels to report consistent condom use, after controlling for sociodemographic and psychosocial variables [8]. These power imbalances, combined with other factors like greater physiological vulnerabilities, contribute to high rates of HIV and other sexually transmitted infections (STIs) [3, 9–12]. While we have known for some time about the potential negative consequences of the intersections between power and sexuality for women, there remains a need to more clearly delineate them among different ages of adolescents and young women (AGYW) in high HIV risk settings. Understanding these nuances will provide better guidance for programs intended to reduce HIV risk and promote SRH. Over the past decade, in fact, influential international institutions like the World Health Organization (WHO) have promoted research and health promotion interventions that address contextual factors like gender-based power imbalances [13].

Methodological challenges related to the quantitative measurement of relationship power had originally inhibited exploration of its impact on health outcomes [2]. In 2000, the Sexual Relationship Power Scale (SRPS) was developed to address the need to measure relationship power in intimate and sexual relationships [14]. The SRPS consists of two subscales measuring the constructs of relationship control (15 items) and decision-making dominance (8 items). Since its development, the SRPS has been used extensively in the field of HIV prevention and sexual risk behavior, and substantial literature exists reporting the psychometric properties of the SPRS and subsequent modifications of the scale. The scale has been translated into over a dozen languages and used with a wide variety of sub-populations (e.g., women and men, young adults and older adults, heterosexual and homosexual populations). Evidence suggests that the core concepts measured within the scale appear to apply globally. At this point, the SRPS is the most widely used tool to measure relationship power in the HIV/STI prevention literature [15]. In a systematic review of the psychometric properties of the scale, the SRPS—and the relationship control (RC) subscale on its own—were found to exhibit sound psychometric properties across multiple study populations and research settings, and to be associated with a variety of key outcomes. The review also found that the scale has been administered to a wide range of ages (e.g., 18 years of age or older, young adults aged 18 to 29, 15 to 49 years old), with the majority including both adolescents and adults in the same sample. Rarely, if ever, had the scale been separately analyzed for different age bands of AGYW.

It is important to understand how young people experience power in sexual relationships at different ages. Many AGYW grow up in hegemonic societies where gender norms—or shared understandings of the appropriate behaviors, roles, and responsibilities of women as opposed to men—reinforce ideals of male strength and control, and female vulnerability and need for protection. Developmental psychologists have demonstrated that messages around gender are transmitted well before adolescence [16]. With the onset of puberty, adolescents are exposed to new expectations from adults and peers that in turn shape their expectations of themselves and those around them; this evolves throughout adolescence and young adulthood [17]. However, it is not clear how these dynamics play out differently in intimate relationships at different stages of adolescence and young adulthood.

Moreover, as adolescence spans nearly a decade (10–19 years old), many health-related behaviors that are influenced by inequitable power in sexual relationship (e.g., who decides if a condom is used) are not common until late adolescence or later. Thus, adolescence presents a window of opportunity for interventions that examine power in sexual relationships and discuss related health behaviors, before these behaviors begin to manifest. Such interventions could influence gender dynamics at a time when adolescent gender socialization processes are under way [18, 19].

Examination of power in young women’s relationships may be critically important in high HIV prevalence contexts, as relationship power may influence a range of HIV-related risk behaviors and experiences. A substantial proportion of AGYW in these contexts are at elevated risk for HIV and other STIs [20]. In the Nyanza region of Kenya (the location of this study), for instance, 5% of adolescent girls (15–19 years) and 14% of young women (20–24 years) are living with HIV [21]. Furthermore, their experience of violence—both having major negative impacts on its own and known to increase risk of HIV acquisition [22]—is high in Kenya; 35% of 15–17-year-olds and 47% of 20–24-year-olds have ever experienced physical or sexual violence [23]. Unprotected sexual activity is a primary and proximal determinant of HIV and other STI acquisition in Kenya, and young women’s inability to negotiate condom use is a key risk factor. Only 3% of 15–19-year-olds and 5% of 20–24-year-old women report current use of the male condom in Kenya [23]. Previous research has shown that women who do not or are unable to communicate with partners or understand their partner’s HIV risk are more likely to be involved in risky sexual behaviors [24, 25]. For instance, while we know that more young women in Kenya are testing and receiving their HIV test results (50% of 15–19-year-olds and 63% of 20–24-year-olds tested in the last 12 months), little is known about young women’s knowledge of their partner’s HIV status. A recent study in Kenya found that only a third of the people who reported testing for HIV, did so with a sexual partner [26]. Similarly, women’s beliefs/awareness of their partner’s sexual concurrency (if the partner had other partners) has been shown to be associated with their increased HIV risk [27]. Yet, we know very little about young women’s awareness of their partner’s sexual networks and how this is influenced by relationship power. In this analysis we validate the SRPS among different age bands of AGYW and examine the associations of relationship power with these key violence and HIV-related health outcomes. We focus on important partner dynamic concerns—including, violence, condom use, knowledge of partner’s HIV status, and beliefs about partner’s concurrency.

## Methods

This study draws upon a cross-sectional survey examining HIV risk and vulnerability among AGYW, aged 15–24 years, residing in Kisumu, Kenya. In this analysis we focus on relationship power amongst different age groups of AGYW and if these are directly associated with HIV-related outcomes. The study was conducted in an urban and a peri-urban community in Kisumu. These communities are part of the U.S President’s Emergency Plan for AIDS Relief (PEPFAR)-supported DREAMS partnership, a program focused on reducing HIV risk and incidence amongst AGYW and their partners. Sites for DREAMS programming were selected by PEPFAR in consultation with Kenyan government representatives and other stakeholders. In general, the study communities are characterized by high poverty levels, high HIV prevalence rates, and a densely populated and/or rapidly growing population. Using the DREAMS program beneficiary rosters and household listings for the program communities prepared by the program implementing partner, we conducted an age-stratified random sample to select potential respondents. Following informed consent procedures, an interviewer-administered survey was conducted by trained female interviewers in a local language of the respondent’s choosing (English, Kiswahili, or Luo) in private convenient locations, usually at a community space used by the DREAMS program or in a private space within/around the respondent’s home. All participants were reimbursed KSH 300 ($2.83 conversion rate on 1 February 2017). The study protocols were reviewed and approved by the Population Council Institutional Review Board, as well by the Kenyatta National Hospital/University of Nairobi Ethics and Research Committee and National Commission for Science Technology and Innovation in Kenya. Written informed consent from study participants (or parental consent and respondent assent, as appropriate) was received from all respondents before proceeding with the survey.

A total of 1,778 AGYW were interviewed for the survey from October 2016 to February 2017. Refusal rate was low; only 20 potential respondents (all aged 20–24 years) refused to participate in the survey, largely due to time constraints. This analysis focuses on AGYW who reported ever having a boyfriend, husband, or partner, even if they had not had sex with this partner (n = 1,101). A comprehensive knowledge, attitudes, and behaviors survey was administered including 16 items addressing power in sexual relationships (drawn from the RC subscale of the SRPS). The SRPS assesses power and control in a sexual relationship (e.g., most of the time, we do what my partner wants to do; my partner tells me who I can spend time with; if I asked my partner to use a condom, he would get angry). Answer choices consist of a 4-point Likert scale (strongly agree to strongly disagree). Scores were calculated using the guidance issued in the original publication describing the development and testing of the SRPS [28]. For each respondent, we calculated the means of the scale by summing each of the items and dividing by the number of non-missing items; note that no items were missing. Higher SRPS scores indicate higher sexual relationship power.

The original RC subscale of the SRPS included 15 items. One of the items (i.e., my partner gets more out of our relationship than I do) was determined by the research team not to translate clearly in the Kenyan setting, so it was dropped, and two new items were developed that were similar in content and considered relevant for AGYW at risk of HIV. During psychometric testing (i.e., confirmatory factor analysis) post survey administration—with the now 16 items—one of the items did not load sufficiently robust and was dropped. Thus, 15 items were used for the power in sexual relationships measure.

Five outcome variables were selected, addressing violence and HIV. The two violence outcomes measured intimate partner violence over the past year (physical and sexual). Questions on physical and sexual partner violence were derived from the WHO’s Violence Against Women Scale, which has been adapted and used in Southern Africa [4, 29]. Questions on partner violence were asked of AGYW who reported having a boyfriend, husband, or partner in the last 12 months. The three HIV risk outcomes include: (1) used condom at last sex with primary partner, (2) partner having other partners in past year (specifically via a question asking “in the last 12 months while you were having a relationship with this person, did this partner have sexual relations/intercourse with persons other than yourself”), and (3) knowledge of partner’s HIV status (created by combining two questions that ask if the partner’s HIV status was known, and then how they came to know it. If the respondent reports that their partner’s status became known via couples testing—which represented >75% of the sample—then this is considered confirmed knowledge of status). Control variables included age, whether they were currently enrolled in school, the last grade completed, and socioeconomic status (constructed using measures of household construction materials, sanitation facilities, and access to water).

Data were analyzed using Stata/IC 14.1. Descriptive analyses were used to describe sociodemographic characteristics and levels of power in sexual relationships. Likelihoods of observed differences in SRPS response items by each age band were examined using Pearson’s chi-squared test for categorical variables. Bivariate and multiple logistic regressions (controlling for sociodemographic characteristics) were applied to assess the associations between sexual relationship power, socio-demographic characteristics, and partner violence and HIV risk outcomes. Among participants aged 15–17 and 18–20, relative odds of the outcomes for each one unit increase in SRPS scores were adjusted to control for “enrolled in school” and socioeconomic status; among participants aged 21–24, odds were adjusted for highest grade completed and socioeconomic status. We did not control for marital status, given the sample all reported having a boyfriend or husband—and marital status is also correlated with education and age—to minimize the likelihood of over-controlling/included variable bias. Note that for the three HIV risk outcomes, the sample included only AGYW who reported they were currently sexually active with their partner (n = 588). Also, when examining relationships with the condom use outcome specifically—as recommended by the SRPS developers—a modified scale was used that removes the three condom related items (for a total of 12 items). Within each age group, collinearity was assessed through fitting a multiple linear regression model of each outcome onto the independent variables to examine variance inflation factors (which all fell below 2.0, indicating they were satisfactory).

Given that the systematic review of SRPS use concluded that it has been successfully used in dozens of studies and the scale should be kept as close to its original form as possible [15], confirmatory factor analysis (CFA) as opposed to exploratory factor analysis (EFA) was used to assess scale performance in this setting. We split respondents into three separate age bands: 15- to 17-, 18- to 20-, and 21- to 24-year-olds, and compared how well the scale functioned for each, as well as compared the two age bands of 15–17 and 18–24-year-olds (since much of the previous validation of the scale had been conducted amongst those 18 years or older). The CFA was conducted using a one-factor model with iterative principal factor estimation using a polychoric correlation matrix. Specifically, examination of scree plots and previous validations of the scale resulted in selection of a one-factor model; an omnibus test indicated there was sufficient evidence to reject the null hypothesis that observed and latent variables were multivariate normal, so iterative principal factor estimation was selected as the best available method; and, because of the possibility of a method effect from using a categorical (Likert) scale to measure a continuous construct (sexual relationship power), a polychoric (as opposed to Pearson) correlation matrix was used. Internal consistency reliability (for all three age bands) was examined via both theta and alpha coefficients, since alphas are quite commonly used for this purpose yet thetas, while similar, more accurately assess reliability for items with answer choices that have less than five options [30].

## Results

### Sample description, and experiences of violence and HIV risk

Respondents (n = 1,101) consisted of 23.7% 15–17-year-olds, 35.4% 18–20-year-olds, and 40.9% 21–24-year-olds. Table 1 presents respondent characteristics across the three different age bands. School enrollment was highest among 15–17-years-olds (70.9%) and lowest among the 21–24-year-olds (19.8%). Fifty-five percent of 15–17-year-olds reported completing some secondary schooling, compared to 61.0% of 18–20-year-olds, and 40.9% of 20–24-year-olds. About one third (34%) of the respondents overall were currently married or living with a man as if married, with substantial differences seen across age group: 2.3% of 15–17-year-olds, 24.9% of 18–20-year-olds, and 56.0% of 20–24-year-olds. A third of the respondents came from households in the lowest socioeconomic quintile across all three age categories. Two thirds (64.9%) of sexually active 15–19-year-olds reported condom use at last sex with their primary partners, compared to half (49.0%) of 18–20-year-olds, and one third (33.5%) of 21–24-year-olds. Approximately 12% of sexually active respondents across the three age bands stated that their partner had other partners in the last year and a high proportion reported that they knew their partner’s HIV status (64.9% of 15–17-year-olds, 81.1% of 18–20-year-olds, and 77.8% of 21–24—year-olds). Violence experience increased with age. More than 10% (11.9%) of 15–17-year-olds, 22.8% of 18–20-year-olds, and almost one third (31.6%) of 21–24-year-olds reported physical violence from intimate partners in the last year. Similarly, nearly 15% (13.8%) of 15–17-year-olds had experienced sexual violence from intimate partners in the last year, compared to 17.4% of 18–20-year-olds and 18.2% of 21–24-year-olds.

**Table 1:** Respondent background characteristics, HIV risk, and experiences of violence.

	Age 15–17	Age 18–20	Age 21–24	
	**N = 1,101 (Total sample)**			
	**(n = 261)**	**(n = 390)**	**(n = 390)**	
**Background characteristics** Enrolled in school				
No	76 (29.1%)	247 (63.3%)	361 (80.2%)	
Yes	185 (70.9%)	143 (36.7%)	89 (19.8%)	
Highest grade completed				
Primary	117 (44.8%)	133 (34.1%)	209 (46.4%)	
Secondary	144 (55.2%)	238 (61.0%)	184 (40.9%)	
Post-secondary	0 (0.0%)	19 (4.9%)	57 (12.7%)	
Socioeconomic status/wealth quintiles				
1 (lowest)	103 (39.5%)	133 (34.1%)	159 (35.3%)	
2	18 (6.9%)	33 (8.5%)	32 (7.1%)	
3	59 (22.6%)	84 (21.5%)	101 (22.4%)	
4	44 (16.9%)	78 (20.0%)	94 (20.9%)	
5 (highest)	37 (14.2%)	62 (15.9%)	64 (14.2%)	
**Marital status**				
Married or living with a man as if married				
Currently	6 (2.3%)	97 (24.9%)	252 (56.0%)	
Formerly	5 (1.9%)	13 (3.3%)	33 (7.3%)	
Never	250 (95.8%)	280 (71.8%)	165 (36.7%)	
**Violence outcomes** Experienced sexual intimate partner violence in last year				
No	225 (86.2%)	322 (82.6%)	368 (81.8%)	
Yes	36 (13.8%)	68 (17.4%)	82 (18.2%)	
Experienced physical intimate partner violence in last year				
No	230 (88.1%)	301 (77.2%)	308 (68.4%)	
Yes	31 (11.9%)	89 (22.8%)	142 (31.6%)	
	**N = 588 (Subsample)**			
	**(n = 57)**	**(n = 206)**	**(n = 325)**	
HIV outcomes*				
Used condom at last sex with primary partner				
No/Don’t know	20 (35.1%)	105 (51.0%)	216 (66.5%)	
Yes	37 (64.9%)	101 (49.0%)	109 (33.5%)	
Believes partner had other partners in last year				
No/Don’t know	50 (87.7%)	180 (87.4%)	284 (87.4%)	
Yes	7 (12.3%)	26 (12.6%)	41 (12.6%)	
Knowledge of partner’s HIV status				
Unconfirmed	20 (35.1%)	39 (18.9%)	72 (22.2%)	
Confirmed	37 (64.9%)	167 (81.1%)	253 (77.8%)	

*Note: HIV outcomes are drawn from sexually active subsample (n = 588).

### Levels of sexual relationship power

Relationship power score means were highest among the younger respondents and decreased by increasing age band among AGYW (2.8, 2.7, and 2.6, respectively; Table 2), meaning that younger respondents reported more power in their sexual relationships than older respondents. These differences were statistically significant (p<0.001, Kruskal-Wallis *H* test).

**Table 2:** Percent of AGYW who agree / strongly agree with individual items measuring power in sexual relationships via the Sexual Relationship Power Scale (SRPS), and mean scale scores (N = 1,101).

Factor	Age 15–17 n = 261	Age 18–20 n = 390	Age 21–24 n = 450	p-value^1^^,^ ^2^
	
1. Most of the time, we do what my partner wants to do.	75 (28.7%)	152 (39.0%)	255 (56.7%)	<0.001
2. My partner won't let me wear certain things.	81 (31.0%)	170 (43.6%)	230 (51.1%)	<0.001
3. When my partner and I are together, I'm pretty quiet.	67 (25.7%)	87 (22.3%)	98 (21.8%)	0.46
4. My partner has more say than I do about important decisions that affect us.	109 (41.8%)	200 (51.3%)	222 (49.3%)	0.049
5. My partner tells me who I can spend time with.	71 (27.2%)	125 (32.1%)	148 (32.9%)	0.26
6. If I asked my partner to use a condom, he would think I'm having sex with other people.	51 (19.5%)	93 (23.8%)	159 (35.3%)	<0.001
7. I feel/felt trapped or stuck in our relationship.	55 (21.1%)	84 (21.5%)	113 (25.1%)	0.34
8. My partner does what he wants, even if I do not want him to.	56 (21.5%)	74 (19.0%)	135 (30.0%)	<0.001
9. I am more committed to our relationship than my partner is.	58 (22.2%)	101 (25.9%)	167 (37.1%)	<0.001
10. When my partner and I disagree, he gets his way most of the time.	85 (32.6%)	138 (35.4%)	206 (45.8%)	<0.001
11. If my partner wants to have sex, he would expect me to agree.	115 (44.1%)	211 (54.1%)	307 (68.2%)	<0.001
12. My partner always wants to know where I am.	146 (55.9%)	258 (66.2%)	297 (66.0%)	0.012
13. If I asked my partner to use a condom, he would get angry.	28 (10.7%)	77 (19.7%)	130 (28.9%)	<0.001
14. If I asked my partner to use a condom, he would get violent.	19 (7.3%)	50 (12.8%)	87 (19.3%)	<0.001
15. My partner might be having sex with someone else.	90 (34.5%)	115 (29.5%)	138 (30.7%)	0.39
16. He lets me know I am not the only partner he could have.	55 (21.1%)	81 (20.8%)	85 (18.9%)	0.71
SRPS Score–mean (SD)	2.823755 (.4007681)	2.744872 (.411995)	2.616389 (.4553935)	<0.001*

^1^Pearson’s chi-squared test for individual items.

^2^ Kruskal-Wallis H test for SRPS means.

We also examined responses to individual SRPS items and found that in general, a substantial proportion of both younger and older AGYW reported limited relationship power. For example, more than half of all respondents agreed or strongly agreed with the statement: “If my partner wants to have sex, he would expect me to agree,” “My partner always wants to know where I am,” and “My partner has more say than I do about important decisions that affect us.” However, when we compared responses to individual items by age band, we again found some consistent differences (Table 2). For example, 11% of 15–17-year-olds, 20% of 18–20-year-olds and 30% of 21–24-year-olds agreed that “If I asked my partner to use a condom, he would get angry” and 33% of 15–17-year-olds, 35% of 18–20-year-olds, and 46% of 21–24-year-olds agreed that “when my partner and I disagree, he gets his way most of the time.” Overall, this held true for 11 out of 16 statements. Findings were also similar when combining respondents into two age bands (15–17 as compared to 18–24), with the older group consistently reporting lower relationship power than the younger group.

### Measurement findings

To understand better how the role of power in sexual relationships is associated with key health outcomes, the individual items (which capture different aspects of relationship power) were combined into one scale (the SRPS). As described above, the SRPS has been widely validated. CFAs confirmed one factor structure for each of the three age bands of AGYW (15–17, 18–20, and 21–24), as well as when split into two age bands (15–17 and 18–24). It was determined that 1 item of the 16 had factor loadings less than .30 and was removed, as is recommended (Table 3) [31]. Overall, alpha coefficients for the resulting 15-item SRPS and 12-item SRPS-M were quite robust (.81 and .76, respectively). The same was found when thetas and alphas were tested for each of the three age bands separately (thetas: .843, .825, and .852, respectively; alphas: .793, .786, and .816).

**Table 3:** Final factor loadings from the confirmatory factor analysis (CFA) for the Sexual Relationship Power Scale (SRPS) items*.

Items	Age 15–17	Age 18–20	Age 21–24
1. Most of the time, we do what my partner wants to do.	0.53	0.52	0.41
2. My partner won't let me wear certain things.	0.36	0.45	0.31
3. When my partner and I are together, I'm pretty quiet.	0.34	0.44	0.50
4. My partner has more say than I do about important decisions that affect us.	0.38	0.45	0.58
5. My partner tells me who I can spend time with.	0.51	0.44	0.50
6. If I asked my partner to use a condom, he would think I'm having sex with other people.	0.75	0.67	0.69
7. I feel/felt trapped or stuck in our relationship.	0.43	0.45	0.39
8. My partner does what he wants, even if I do not want him to.	0.57	0.57	0.63
9. I am more committed to our relationship than my partner is.	0.40	0.42	0.44
10. When my partner and I disagree, he gets his way most of the time.	0.55	0.57	0.67
11. If my partner wants to have sex, he would expect me to agree.	0.50	0.35	0.38
12. My partner always wants to know where I am.	0.29	0.32	0.24
13. If I asked my partner to use a condom, he would get angry.	0.66	0.62	0.70
14. If I asked my partner to use a condom, he would get violent.	0.52	0.55	0.66
15. My partner might be having sex with someone else.	0.49	0.33	0.44
16. He lets me know I am not the only partner he could have.	0.58	0.40	0.45

*One item with factor loadings < .30 (q12) was dropped from final scales for all age bands.

Note: Coefficient thetas for final 15-item scale for ages 15–17, 18–20, and 21–24, respectively, were: .843, .825, and .852.

### Associations amongst key outcomes, sociodemographic factors, and relationship power

We examined the bivariate relationships between sociodemographic characteristics (i.e., whether respondent was enrolled in school, highest grade completed, and socioeconomic status), relationship power, and our five key outcomes related to partner violence and HIV risk, for each of the three age bands (Table 4). For 15–17-year-olds, higher sexual relationship power was consistently associated with outcomes, including condom use at last sex (OR 4.30), whether the partner had other partners (OR 0.06), sexual partner violence (OR 0.16), and physical partner violence (OR 0.18). Being enrolled in school was also associated with condom use at last sex (OR 11.25) and whether the partner’s HIV status was known (OR 0.29), for 15–17-year-olds. For 18–20-year-olds, relationship power was associated with sexual violence, physical violence, and whether the partner had other partners. Schooling (i.e., enrolment in school or highest grade completed) was associated with condom use at last sex, whether the partner had other partners, and physical violence, for 18–20-year-olds. For the oldest group (21–24-year-olds), relationship power was associated with whether the partner had other partners, knowledge of partner’s HIV status, sexual violence, and physical violence. Schooling (i.e. enrollment in school or highest grade completed) was associated with condom use at last sex, knowledge of partner’s HIV status, and physical violence, for 21–24-year olds. Socio-economic status was not associated with any of the key outcomes, for any age band.

**Table 4:** Bivariate associations between HIV and partner violence outcomes, and sexual relationship power (SRPS or SRPS-M) and sociodemographic characteristics.

		Age 15–17			Age 18–20			Age 21–24		
		OR	p-value	95% CI	OR	p-value	95% CI	OR	p-value	95% CI
Used condom at last sex with primary partner*	Enrolled in school	11.25	< .001	(4.75, 26.62)	4.37	< .001	(2.31, 8.27)	5.87	0.007	(1.64, 21.04)
	Highest grade completed	4.59	< .001	(2.62, 8.05)	1.32	0.098	(0.95, 1.82)	4.22	0.026	(1.18, 15.05)
	Socioeconomic status	1.03	0.738	(0.86, 1.25)	0.97	0.694	(0.82, 1.14)	1.03	0.861	(0.71, 1.5)
	SRPS-M score	4.30	0.000	(1.99, 9.32)	1.21	0.474	(0.72, 2.06)	1.77	0.465	(0.38, 8.25)
**Partner had other partners in last year**	Enrolled in school	0.17	0.109	(0.02, 1.49)	0.87	0.786	(0.33, 2.31)	0.57	0.314	(0.2, 1.69)
	Highest grade completed	0.55	0.502	(0.1, 3.13)	0.34	0.012	(0.14, 0.78)	0.60	0.060	(0.36, 1.02)
	Socioeconomic status	0.64	0.173	(0.34, 1.21)	0.91	0.523	(0.69, 1.21)	1.15	0.223	(0.92, 1.45)
	SRPS score	0.06	0.038	(0, 0.86)	0.07	< .001	(0.02, 0.22)	0.09	< .001	(0.04, 0.21)
**Partner’s HIV status known (via couple testing)**	Enrolled in school	0.29	0.034	(0.09, 0.91)	1.16	0.730	(0.51, 2.63)	1.55	0.289	(0.69, 3.47)
	Highest grade completed	1.02	0.968	(0.34, 3.1)	1.59	0.158	(0.84, 3.02)	1.65	0.017	(1.09, 2.48)
	Socioeconomic status	0.96	0.840	(0.67, 1.39)	0.94	0.639	(0.74, 1.2)	0.95	0.580	(0.79, 1.14)
	SRPS score	0.96	0.959	(0.21, 4.44)	1.44	0.408	(0.61, 3.38)	2.44	0.003	(1.36, 4.38)
**Experienced sexual intimate partner violence in last year**	Enrolled in school	1.08	0.849	(0.49, 2.36)	0.86	0.593	(0.5, 1.49)	0.72	0.325	(0.38, 1.38)
	Highest grade completed	1.02	0.960	(0.5, 2.07)	0.55	0.015	(0.34, 0.89)	0.74	0.101	(0.51, 1.06)
	Socioeconomic status	1.16	0.203	(0.92, 1.47)	1.08	0.375	(0.91, 1.29)	1.16	0.079	(0.98, 1.36)
	SRPS score	0.16	< .001	(0.07, 0.4)	0.15	< .001	(0.08, 0.3)	0.08	< .001	(0.04, 0.16)
**Experienced physical intimate partner violence in last year**	Enrolled in school	0.72	0.408	(0.33, 1.58)	0.45	0.004	(0.26, 0.78)	0.41	0.003	(0.23, 0.73)
	Highest grade completed	1.55	0.268	(0.71, 3.39)	0.59	0.017	(0.38, 0.91)	0.56	0.000	(0.41, 0.77)
	Socioeconomic status	0.94	0.662	(0.73, 1.22)	0.99	0.944	(0.85, 1.17)	1.04	0.565	(0.91, 1.19)
	SRPS score	0.18	0.000	(0.07, 0.46)	0.16	< .001	(0.09, 0.31)	0.16	< .001	(0.09, 0.26)

*Note: SRPS-M (N = 12 items, removing the 3 condom related questions from the 15-item SRPS) used in association with condom use at last sex outcome. All other results shown use 15-item SRPS score.

To clarify the role of relationship power *vis-à-vis* the five outcomes, multiple logistic regression models were fit for each of the five partner violence and HIV risk outcomes on power in sexual relationships as measured by the SRPS (Table 5). Levels of relationship power predicted key outcomes for each age band of AGYW, when controlling for schooling and socioeconomic status. Among 15–17-year-olds, AGYW with higher relationship power (i.e., higher mean score on the SRPS), reported decreased likelihood of sexual (aOR 0.13, 95% CI: 0.05–0.33, p<0.001) and physical (aOR 0.18, 95% CI: 0.07, 0.47, p<0.001) violence from intimate partners in the last year, compared to 15–17-year-olds with lower relationship power. This equates to seven times less likely to experience sexual partner violence, and more than five times less likely to experience physical partner violence. For 18–20-year-olds, those with higher relationship power reported decreased likelihood of sexual (aOR 0.15, 95% CI: 0.08, 0.30, p<0.001) and physical (aOR 0.18, 95% CI: 0.09, 0.33, p<0.001) violence from intimate partners in the last year, and increased likelihood of condom use at last sex (aOR 3.75, 95% CI: 1.60, 8.81, p<0.01) compared to 18–20-year-olds with lower relationship power. This equates to six times less likely to experience sexual partner violence, over five times less likely to experience physical partner violence, and almost four times more likely to use a condom. For 21–24-year-olds, those with higher relationship power reported decreased likelihood of sexual (aOR 0.08, 95% CI: 0.04, 0.15, p<0.001) and physical (aOR 0.17, 95% CI: 0.10, 0.29, p<0.001) violence from intimate partners in the last year, and partner having other partners in the last year (aOR 0.10, 95% CI: 0.04, 0.22, p<0.001), and increased likelihood of knowledge of partner’s HIV status (aOR 2.06, 95% CI: 1.10, 3.84, p<0.05), compared to 21–24-year-olds with lower relationship power. This equates to more than 12 times less likely to experience sexual partner violence, and over 5 times less likely to experience physical partner violence, among the other findings.

**Table 5:** Adjusted odds ratios for HIV and partner violence outcomes on Sexual Relationship Power Scale (SRPS) scores, stratified by AGYW age band.

Age	Used condom at last sex with primary partner^1^ aOR (95% CI)	Partner had other partners in last year aOR (95% CI)	Partner’s HIV status known via couple testing aOR (95% CI)	Experienced sexual intimate partner violence in last year aOR (95% CI)	Experienced physical intimate partner violence in last year aOR (95% CI)
15–17^2^	2.34 (0.41, 13.38)	0.03 (< .001, 1.03)	0.89 (0.18, 4.41)	0.13 (0.05, 0.33)***	0.18 (0.07, 0.47)***
18–20^2^	3.75 (1.60, 8.81)**	0.05 (0.02, 0.18)***	1.38 (0.57, 3.34)	0.15 (0.08, 0.30)***	0.18 (0.09, 0.33)***
21–24^3^	1.04 (0.59, 1.82)	0.10 (0.04, 0.22)***	2.06 (1.10, 3.84)*	0.08 (0.04, 0.15)***	0.17 (0.10, 0.29)***

^1^Condom use outcome applies 12-item SRPS-M score, removing the 3 condom-related SRPS items. All other results shown use 15-item SRPS score.

^2^Controlled / adjusted for: enrolled in school, socioeconomic status.

^3^Controlled / adjusted for: highest grade completed, socioeconomic status.

*p<0.05

**p<0.01

***p<0.001

## Discussion/Conclusions

The sexual and intimate relationships of 15–24-year-old respondents in Kisumu, Kenya were characterized by having limited interpersonal power vis-à-vis their partners, with a range of controlling partner behaviors reported. Further, low relationship power was an important predictor of HIV risk and partner violence, even when taking in account schooling and socioeconomic status. The strength of the associations with sexual and physical partner violence was particularly notable. There was a consistent association between low relationship power and sexual, and physical, violence, for each of the three age bands within the 15–24-year-old range (i.e., 15–17, 18–20, and 21–24). A strong link between the SRPS and partner violence is also reflected in numerous other studies across sub-Saharan Africa and locations across the globe [15]. The associations between relationship power and the three HIV risk variables were also important, but less consistent. When controlling for sociodemographic characteristics, power was associated with condom use, and with knowledge of partner’s HIV status, for one age band but not the other two, and with awareness of their partner having other sexual partners for two age bands but not the third. However, another analysis with this sample of AGYW has demonstrated that sexual violence was associated with poor HIV outcomes—including increased STI experience and increased HIV risk perception—as well as depression and anxiety [32]. Thus, addressing relationship power (as measured by the SRPS) could have an important impact on addressing vulnerability to HIV via its association with violence. Further, prior studies have in fact commonly demonstrated an association between the SRPS and condom use, whereby lower relationship power was associated with a lack of condom use, or more inconsistent condom use [15]. For the current study, it may also be that the relatively small sample of younger (15–17-year-olds) sexually active adolescents constrained our ability to measure significant associations; younger adolescents with more relationship power were twice as likely to report condom use—even after controls were taken into account—but this difference did not achieve statistical significance.

This study also sheds new light on how different age groups within the 15–24-year-old age range can experience power in sexual relationships differently. While many assume that younger girls tend to have less power vis-à-vis their partners than their older counterparts (due to factors such as having less well-developed negotiation skills), we found that women over 18 years old reported less power in their sexual relationships than did girls under 18 years old. This finding could be explained by the fact that many of the older girls were married and may well have had less negotiating power in a marital relationship than a younger girl who could more easily leave the relationship in question. Alternatively, these types of questions may have less salience for a younger girl who has not yet started sexual relationships; however, all respondents did report a boyfriend/partner, and the controlling behaviors described in the SRPS (e.g., my partner always wants to know where I am) generally are not dependent on whether the relationship includes sexual activity. Either way, we need to become more nuanced in our understanding of and response to these dynamics, to segment our audiences appropriately and to tailor our programmatic approaches to specific age ranges [33]. And, future research comparing not only subsets of AGYW among 15–24-year-olds, but also women across the life span in a given community, as well as the effects of age disparate relationships, would be helpful to provide additional insights around how relationship power may differ for different age groups.

Moreover, the Sexual Relationship Power Scale was demonstrated to be a good measure for relationship power across the sample, including the 15–17, 18–20, and 21–24-year-old age bands. As programs for AGYW are increasingly tailored to meet their needs, such as by explicitly including a relationship power component, it is useful to draw upon evaluation measures that have been tested and adapted for this specific population. We note that the magnitude of the relationships with the SRPS found in this study were quite high (e.g., high odds ratios). This is a common finding with the scale (as small shifts in score seem to indicate substantial shifts in relationship power).

Certain limitations to the study should be highlighted. We rely on cross sectional data here and are thus unable to tease apart causal relationships between power in sexual relationships and HIV and partner violence outcomes. However, these data are drawn from an ongoing implementation science study, and we plan to further explore these relationships in the future. We also rely on self-reported data, which can be affected by social desirability biases. For instance, only about half of the AGYW in our sample reported that they had ever had sex with their intimate partners which may be limiting our ability to examine power within AGYW relationships.

Our findings highlight the need for increased efforts to address unequal power dynamics in relationships, so that we can stem experiences of partner violence and HIV acquisition for AGYW. This finding is consistent with a comprehensive review of HIV and sexuality education evaluations which found that programs addressing power or gender were five times as likely to be effective as those that did not [34]. Fully 80% of those programs were associated with a significantly lower rate of STIs or unintended pregnancy, while in contrast, among the programs that did not address gender or power, only 17% had such an association.

There is an urgent need for the implementation (or adaptation) of new and innovative approaches to break the cycle of high HIV incidence amongst and violence against AGYW [35]. Varied programs intended to achieve this goal are currently being rolled out and evaluated. Common implementation strategies either focus on targeted approaches (e.g., couples counseling [36]) or comprehensive approaches (e.g., reaching a combination of key stakeholders and creating an enabling environment for AGYW empowerment [37]). The DREAMS partnership—which stands for Determined, Resilient, Empowered, AIDS-free, Mentored, and Safe, and is currently taking place in Kenya and other sub-Saharan African countries—applies a comprehensive approach to reducing HIV acquisition amongst AGYW. Empowering young women is a primary goal of DREAMS, within a context of also influencing stakeholders that surround AGYW, such as parents, community leaders, health providers, and their sexual partners. Ongoing studies are examining the lessons learned from these approaches and the effects of the DREAMS program [38, 39].

In most of sub-Saharan Africa, AGYW bear a disproportionate burden of HIV risk and this is coupled with high prevalence of physical and sexual partner violence. Our findings suggest that inequitable power in sexual relationships contributes in important ways to these challenges for AGYW. Going forward, these power dynamics should be addressed explicitly in programs, and relationship power should be measured explicitly in evaluations—in addition to the HIV and violence outcome measures—to determine whether these dynamics are shifting.

## References

[1] Amaro H. Love, sex, and power: considering women's realities in HIV prevention. Am Psychol. 1995;50: 437–447. 7598292 10.1037//0003-066x.50.6.437

[2] Blanc AK. The effect of power in sexual relationships on sexual and productive health: An examination of the evidence. Stud Fam Plann. 2001;32: 189–213. 11677692 10.1111/j.1728-4465.2001.00189.x

[3] Campbell AN, Tross S, Dworkin SL, Hu MC, Manuel J, Pavlicova M, et al Relationship power and sexual risk among women in community-based substance abuse treatment. J Urban Health. 2009;86(6): 951–964. 10.1007/s11524-009-9405-0 19921541 PMC2791824

[4] Jewkes RK, Dunkle K, Nduna M, Jama Shai NP. Intimate partner violence, relationship power inequity, and incidence of HIV infection in young women in South Africa: A cohort study. The Lancet. 2010;376(9734): 41–48. 10.1016/S0140-6736(10)60548-X 20557928

[5] Connell RW. Gender and power Stanford, CA: Stanford University Press; 1987.

[6] Hatcher AM, Tsai AC, Kumbakumba E, Dworkin SL, Hunt PW, Martin JN, et al Sexual relationship power and depression among HIV-infected women in rural Uganda. PLoS One. 2012;7(12): e49821 10.1371/journal.pone.0049821 23300519 PMC3530575

[7] Wingood G, DiClemente R. Partner influences and gender-related factors associated with noncondom use among young adult African-American women. Am J Community Psychol. 1998;26: 29–51. 9574497 10.1023/a:1021830023545

[8] Pulerwitz J, Amaro H, DeJong W, Gortmaker SL, Rudd R. Relationship power, condom use, and HIV risk among women in the USA. AIDS Care. 2002;14(6): 789–800. 10.1080/0954012021000031868 12511212

[9] Glynn JR, Caraël M, Auvert B, Kahindo M, Chege J, Musonda R, et al Why do young women have a much higher prevalence of HIV than young men? A study in Kisumu, Kenya and Ndola, Zambia. AIDS. 2001;15: S51–S60. 11686466 10.1097/00002030-200108004-00006

[10] Leclerc-Madlala S. Age-disparate and intergenerational sex in southern Africa: The dynamics of hypervulnerability. AIDS. 2008;22: S17–S25. 10.1097/01.aids.0000341774.86500.53 19033752

[11] Higgins JA, Hoffman S, Dworkin SL. Rethinking gender, heterosexual men, and women's vulnerability to HIV/AIDS. Am J Public Health. 2010;100: 435–445. 10.2105/AJPH.2009.159723 20075321 PMC2820057

[12] Mavedzenge SN, Weiss HA, Montgomery ET, Blanchard K, de Bruyn G, Ramjee G, et al Determinants of differential HIV incidence among women in three southern African locations. J Acquir Immune Defic Syndr. 2011;58(1): 89–99. 10.1097/QAI.0b013e3182254038 21654502

[13] World Health Organization. Woman and health: today’s evidence, tomorrow’s agenda Geneva, Switzerland: World Health Organization; 2009.

[14] Pulerwitz J, Gortmaker SL, Dejong W. Measuring relationship power in HIV/STD research. Sex Roles. 2000;42: 637–660.

[15] McMahon JM, Volpe EM, Klostermann K, Trabold N, and Xue Y. A systematic review of the psychometric properties of the Sexual Relationship Power Scale in HIV/AIDS research. Arch Sex Behav. 2015;44(2): 267–294. 10.1007/s10508-014-0355-6 25331613 PMC4324007

[16] Martin CL, Ruble DN, Szkrybalo J. Cognitive theories of early gender development. Psychol Bull. 2002;128(6): 903–933. 12405137 10.1037/0033-2909.128.6.903

[17] Lundgren R, Burgess S, Chantelois H, Oregede S, Kerner B and Kågesten AE. Processing gender: lived experiences of reproducing and transforming gender norms over the life course of young people in Northern Uganda. Cult Health Sex. 2018; 8 6:1–17 (epub ahead of print). 10.1080/13691058.2018.1471160 29882476

[18] Blum RW, Astone NM, Decker MR, Mouli VC. A conceptual framework for early adolescence: a platform for research. Int J Adolesc Med Health. 2014;26: 321e31 10.1515/ijamh-2013-0327 24486726 PMC4476282

[19] Igras SM, Macieira M, Murphy E, Lundgren R. Investing in very young adolescents’ sexual and reproductive health. Glob Public Health. 2014;9: 555e69 10.1080/17441692.2014.908230 24824757 PMC4066908

[20] UNAIDS. Prevention gap report. Geneva: Joint United National Programme on HIV/AIDS (UNAIDS); 2016.

[21] National AIDS and STI Control Programme (NASCOP), Kenya. Kenya AIDS Indicator Survey 2012: Final Report. Nairobi: NASCOP; 2014.

[22] Li Y, Marshall CM, Rees HC, Nunez A, Ezeanolue EE, Ehiri JE. Intimate partner violence and HIV infection among women: a systematic review and meta-analysis. J Int AIDS Soc. 2014;17(1): 18845 10.7448/IAS.17.1.18845 24560342 PMC3925800

[23] KNBS and ICF International. Kenya demographic and health survey 2014 Calverton: KNBS and ICF International; 2015.

[24] Sayles JN, Pettifor A, Wong MD, MacPhail C, Lee SJ, Hendriksen E, et al Factors associated with self-efficacy for condom use and sexual negotiation among South African youth. J Acquir Immune Defic Syndr. 2006;43(2): 226–33. 10.1097/01.qai.0000230527.17459.5c 16951647 PMC2819666

[25] Higgins JA, Mathur S, Eckel E, Kelly L, Nakyanjo N, Sekamwa R, et al Importance of relationship context in HIV transmission: results from a qualitative case-control study in Rakai, Uganda. American Journal of Public Health. 2014;104(4): 612–20. 10.2105/AJPH.2013.301670 24524490 PMC4025686

[26] Kimanga DO, Ogola S, Umuro M, Ng’ang’a A, Kimondo L, Murithi P, et al Prevalence and incidence of HIV infection, trends, and risk factors among persons aged 15–64 years in Kenya: results from a nationally representative study. J Acquir Immune Defic Syndr. 2014;66(Suppl 1): S13–26. 10.1097/QAI.0000000000000124 24445338 PMC4794992

[27] Mah TL, Shelton JD. Concurrency revisited: increasing and compelling epidemiological evidence. J Int AIDS Soc. 2011;14(1): 33 10.1186/1758-2652-14-33 21689437 PMC3133533

[28] Pulerwitz J, Gortmaker S, and DeJong J. Measuring sexual relationship power in HIV/STD research. Sex Roles. 2000;42(7–8): 637–660.

[29] Gibbs A, Washington L, Willan S, Ntini N, Khumalo T, Mbatha N, et al The Stepping Stones and Creating Futures intervention to prevent intimate partner violence and HIV-risk behaviours in Durban, South Africa: study protocol for a cluster randomized control trial, and baseline characteristics. BMC Public Health. 2017;17(1): 336 10.1186/s12889-017-4223-x 28427380 PMC5397780

[30] Zumbo B, Gadermann A, Zeisser C. Ordinal versions of coefficient alpha and theta for Likert scale. J Mod Appl Stat Methods 2007;6(1): 21–9.

[31] Hair JFJ, Tatham RL, Anderson RE, Black W. Multivariate data analysis 5th ed. Upper Saddle River, NJ: Prentice Hall; 1998.

[32] Mathur S, Okal J, Musheke M, Pilgrim N, Patel SK, Bhattacharya R, et al High rates of sexual violence by both intimate and non-intimate partners experienced by adolescent girls and young women in Kenya and Zambia: Findings around violence and other negative health outcomes. PLOS One; under review. 10.1371/journal.pone.0203929 PMC6136792 30212561

[33] Bruce J. Commentary: Investing in the poorest girls in the poorest communities early enough to make a difference. Global Public Health 2015;10(2): 225–227. 10.1080/17441692.2014.986170 25555080 PMC4318008

[34] Haberland N. The case for addressing gender and power in sexuality and HIV education: a comprehensive review of evaluation studies. Int Perspect Sex Reprod Health. 2015,41(1): 31–42. 10.1363/4103115 25856235

[35] Dunkle KL, Jewkes R, Brown HC, Gray GE, McIntryre JA, Harlow SD. Gender-based violence, relationship power, and risk of HIV infection in women attending antenatal clinics in South Africa. The Lancet. 2004;363(9419): 1415–1421. 10.1016/S0140-6736(04)16098-4 15121402

[36] Haberland N, Ndwiga C, McCarthy K, Pulerwitz J, Kosgei R, Mak’anyengo M, et al. Addressing intimate partner violence in HIV testing services: positive results of a randomized controlled trial in Nairobi, Kenya [poster]. International AIDS Society Conference 2017; 24 July 2017; Paris, France.

[37] Campbell C, Cornish F. Towards a “fourth generation” of approaches to HIV/AIDS management: creating contexts for effective community mobilization. AIDS Care. 2010;22(Sup 2): 1569–79. 10.1080/09540121.2010.525812 21161761

[38] Population Council. How to best reduce HIV risk among adolescent girls and young women in sub-Saharan Africa?: implementation science around the DREAMS Partnership, DREAMS Project Brief. Washington, DC: Population Council; 2016.

[39] London School of Hygiene & Tropical Medicine. Study protocol: impact evaluation of PEPFAR’s DREAMS initiative in 4 sites London: London School of Hygiene & Tropical Medicine; 2017 Available from: https://www.lshtm.ac.uk/files/DREAMS_IE_Multi-Country_Protocol_2017.pdf

